# Spin-related tunneling through a nanostructured electric-magnetic barrier on the surface of a topological insulator

**DOI:** 10.1186/1556-276X-7-90

**Published:** 2012-01-27

**Authors:** Zhenhua Wu, Jun Li

**Affiliations:** 1 SKLSM, Institute of Semiconductors, Chinese Academy of Sciences, P.O. Box 912, Beijing 100083, China; 2 CAE Team, Semiconductor R&D Center, Samsung Electronics Co., Ltd., Gyeonggi-Do, Korea; 3 Department of Physics, Semiconductor Photonics Research Center, Xiamen University, Xiamen 361005, China

## Abstract

We investigate quantum tunneling through a single electric and/or magnetic barrier on the surface of a three-dimensional topological insulator. We found that (1) the propagating behavior of electrons in such system exhibits a strong dependence on the direction of the incident electron wavevector and incident energy, giving the possibility to construct a wave vector and/or energy filter; (2) the spin orientation can be tuned by changing the magnetic barrier structure as well as the incident angles and energies.

PACS numbers: 72.25.Dc; 73.20.-r; 73.23.-b; 75.70.-i.

## 1. Introduction

The recent discovery of a new quantum state of matter, topological insulator, has generated a lot of interest due to its great scientific and technological importance [1-5]. In a topological insulator, spin-orbit coupling opens an energy gap in the bulk, and results in helical surface states residing in the bulk gap in the absence of magnetic fields. Such surface states are spin-dependent and are topologically protected by time-reversal symmetry [4-7] and distinct from conventional surface states, which are fragile and depend sensitively on the details of the surface geometry and bonding. This discovery sparked intensive experimental and theoretical interests, both for its fundamental novel electronics properties as well as possible applications in a new generation of electric devices.

Very recently, the surface states in Bi-based alloys, Bi_1-*x*_Sb_*x*_, Bi_2_Se_3_, Bi_2_Te_3_, were theoretically predicted [6,8] and experimentally observed by using angle-resolved photoe-mission spectroscopy (ARPES) [9-12]. These 3D topological insulators have robust and simple surface states consisting of a single Dirac cone at the Γ point [8]. Note that this Hamiltonian appears similar to graphene [13], but topological insulators have an odd number of massless Dirac cones on the surface, ensured by the *Z*_2 _topological invariant of the bulk, while graphene has twofold massless Dirac cones at the *K *and *K' *valleys. Another essential difference is connected with spin-related properties. In the surface, Hamiltonian of the 3D TI ***σ ***acts on the real spin of the charge carriers, while for graphene it stands for the pseudo spin, i.e., the A and B sublattices of graphene. Hence, it is natural to manipulate spin transport on the surface of a 3D topological insulator by controlling the electron orbital motion. Based on the topological surface Hamiltonian, it is clear that ***σ ***· ***k ***is a quantum conserved quantity which implies that spin and momentum of the electron are locked. For instance in a tunneling process, the reflected electron will reverse its spin due to the helical property of the surface states, i.e., the spin-momentum locking [14]. This feature will lead to some interesting phenomena, such as the spin-dependent conductance [15] and the twisted RKKY interaction [16].

In this study, we investigate electron tunneling through single electric and magnetic potential barriers which can be created by depositing a ferromagnetic metallic strip on the surface of a 3D topological insulator. We find that the in-plane spin orientation of the transmitted and the reflected electrons can be rotated over certain angles that are determined by the incident angle and energy. Our results demonstrate that the magnetic field of the magnetic barrier bends the trajectory of the electrons, and therefore rotate the spin.

## 2. Theory

**Figure 1:**
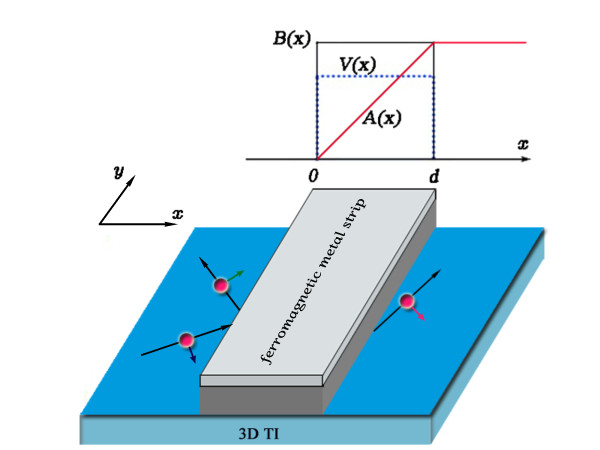
**Schematic of an electric and magnetic potential barrier on the surface of a 3D TI**.

First we focus on the electron transmission through a single electric and magnetic potential barrier on the surface of a 3D topological insulator, as shown in Figure 1. We can create magnetic and electric potentials underneath a superconducting plate and a ferromagnetic strip [17-20]. The relevant magnetic and electric fields are directed perpendicular to the surface of the 3D TI, i.e., *V*(*x*) = *V*[Θ(*x*) - Θ(*x *- *d*)]/2 and *B*_*z*_(*x*) = *B*[Θ(*x*) - Θ(*x *- *d*)]/2, where Θ is the Heaviside step function. The magnetic field is perpendicular to the surface and the Landau gauge ***A ***= (0, *Bx*, 0) is adopted in our calculation. The low-energy electrons near the Γ point of the Dirac cones can be well described by the effective Hamiltonian [21],

(1) $$H=v_{\text{F}}\sigma \cdot \left(\pi \times \hat{z}\right)+V+H_{z},$$

where *v*_F _is the Fermi velocity, *σ*^*i*^(*i *= *x, y, z*) are the Pauli matrices, *V *is the gate voltage applied on the magnetic metal strips, and the last term *H*_*Z *_≡ *gμ*_*B*_*σ *· ***B ***is induced by Zeeman spin spitting. Note that for *g *= 23 in Bi_2_Se_3_, the Zeeman term affects the transmission slightly at low magnetic field. The momentum is *π *= **p **+ **e*A***, where the vector potential of the inhomogeneous magnetic field generated by the magnetic metal stripe, $\hat{z}$, is the unit vector normal to the surface. Note that $\left[\sigma \cdot \left(\pi \times \hat{z}\right),H\right]=0$, which implies that $\sigma \cdot \left(\pi \times \hat{z}\right)$ is a quantum conserved quantity during the tunneling processes, i.e., spin-momentum locking. If the incident electrons are spin polarized along the direction of the vector $\left(\pi \times \hat{z}\right)$, the magnetic field bends the trajectory of the electrons, resulting in a rotation of the spin of the transmitted electrons. The reflected electrons suffer similar spin rotations accompanied by the reversal of the momentum *p*_*x*_. Interestingly, the gate voltage can be used to control the reflection and transmission, and therefore tune the spin polarization of the reflected and transmitted electrons.

For simplicity, we introduce the dimensionless units: *l*_*B *_= [*ħ*/*eB*_0_]^1/2^, *B*(*x*) → *B*_0_*B*(*x*), *A*_*y*_(*x*) → *B*_0_*l*_*B*_*A_y_*(*x*), *E *→ *E*_0_*E*, ***r ***→ *l*_*B*_***r***, ***k ***→ ***k***/*l*_*B*_, and rewrite the Hamiltonian as

(2) $$H=\left(\begin{array}{cc} V+g\mu_{B}B & k_{y}+A_{y}+ik_{x}\\ k_{y}+A_{y}-ik_{x} & V-g\mu_{B}B \end{array}\right).$$

In the presence of the magnetic-electric potentials, the wavevector satisfies $k_{x}^{2}+{\left(k_{y}+A_{y}\right)}^{2}={\left(E-V\right)}^{2}-{\left(g\mu_{B}B\right)}^{2}$. Note that the translational invariance along the *y *direction gives rise to conservation of *k*_*y*_, and thus the solutions can be written as $\psi \left(x,y\right)=\psi \left(x\right)e^{ik_{y}y}$. In the free region, the eigenvalues are *E*_± _= ±*v*_*F*_*π*, and the corresponding eigenvectors are

(3) $$\begin{array}{ll} \psi_{L}\left(x\right) & =\frac{1}{\sqrt{2}}\left(\begin{array}{c} 1\\ \frac{-ik_{x}+\left(k_{y}+A_{y}\right)}{E_{F}-V} \end{array}\right)e^{ik_{x}x}\;\\ & \;+\frac{r}{\sqrt{2}}\left(\begin{array}{c} 1\\ \frac{ik_{x}+\left(k_{y}+A_{y}\right)}{E_{F}-V} \end{array}\right)e^{-ik_{x}x},\;\\ & \; \end{array}$$

(4) $$\psi_{R}\left(x\right)=\frac{t}{\sqrt{2}}\left(\begin{array}{c} 1\\ \frac{-ik_{x}+\left(k_{y}+A_{y}\right)}{E_{F}-V} \end{array}\right)e^{ik_{x}x}.$$

In the barrier region the vector potential is ***A ***= (0, *Bx*, 0) and the solution can be expressed in terms of parabolic cylinder functions *D*_*ν*_,

(5) $$\psi =\underset{\pm }{\sum }c_{\pm }\left(\begin{array}{c} D_{v/2-1}\left[\pm \sqrt{2}\left(x+k_{y}\right)\right]\\ \pm \sqrt{\frac{2}{v}}D_{v/2}\left[\pm \sqrt{2}\left(x+k_{y}\right)\right] \end{array}\right),$$

with complex coefficients *c*_± _and *υ *≡ (*E*_*F *_- *V*)^2 ^- (*gμ*_*B*_*B*)^2^. After some lengthy algebra, all of the above coefficients of the wave functions can be obtained from the boundary conditions.

## 3. Spin and momentum filtering

**Figure 2:**
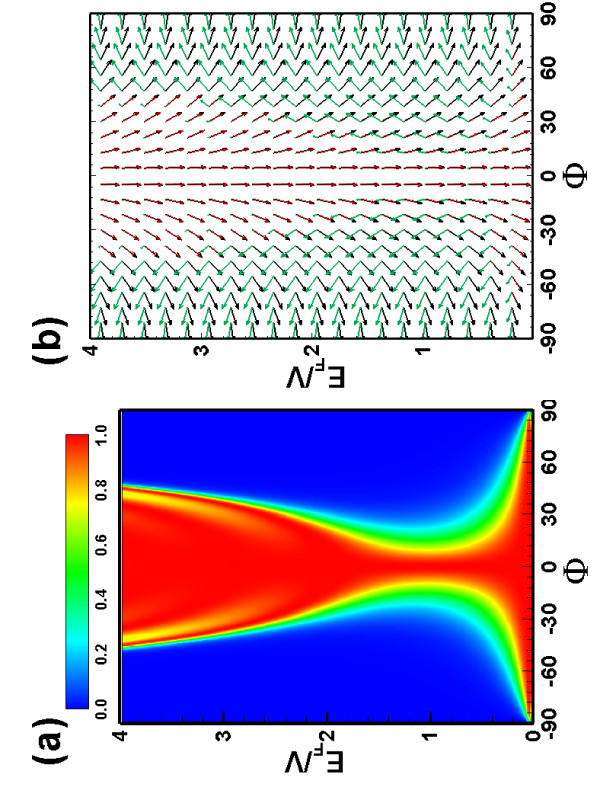
**Transmission spectrum through the single magnetic/electric barrier and spin rotation of transmitted/reflected electrons**. **(a) **The contour plot of the transmission probability as a function of the incident angle and the incident energy, for a fixed barrier width *d *= 1 and height *V *= 4, for magnetic field *B *= 0. **(b) **The spin orientation of the incident (black arrows), transmitted (red arrows), and reflected (green arrows) electrons as a function of the incident angle and energy. The lengths of the arrows indicate the transmitted or reflected probabilities. The magnetic field unit is *B*_0 _= 1 *T*, the energy unit is *E*_0 _= 16 meV, and the length unit is *l*_*B *_= 26 nm.

First we consider that the incident electrons are spin polarized along the direction of the vector $\left(\pi \times \hat{z}\right)$, i.e., perpendicular to the direction of the electron motion. We examine the transmission characteristics for a pure electric barrier (*B *= 0, *V *= 4), which are shown in Figure 2a,b. In Figure 2a we plot the transmission probability as a function of the incident angle and energy. The symmetry of the low-energy effective Hamiltonian when ***A ***= 0 ensures the invariance of the transmission with respect to *k*_*y *_→ -*k*_*y*_. Note that, perfect transmission always exists in the vicinity of normal incidence, i.e., this is the so-called Klein tunneling, induced by the helical property of the Dirac fermion. This perfect tunneling process can even occur for a low incident energy, and a high and wide barrier. Figure 2b shows the spin orientation as a function of the incident angles and the incident energies. From the spin orientation one can see that the transmitted electron spins are polarized along the same direction as the incident electron spin, indicating that a pure electric barrier without magnetic field will not affect the spin orientation, the reason is that an electric barrier cannot bend the trajectories of the electrons and the outgoing electrons will propagate in the same direction as the incident ones. The spin of the reflected electron, with reversed longitudinal wavevector and conserved transverse wavevector, is rotated due to the spin-momentum locking.

**Figure 3:**
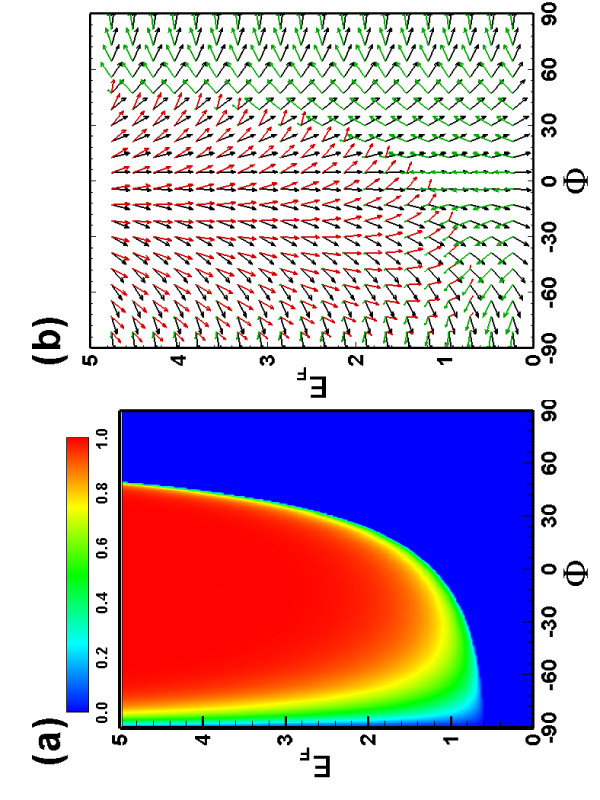
**The same as Figure 2, but now for a pure magnetic barrier with *B *= 1, and *V *= 0**.

Next we consider the tunneling process through a pure magnetic single barrier. The transmission probability is shown in Figure 3a for a magnetic barrier (*B *= 1) with width *d *= 1. Compared to the pure electric barrier case (see Figure 2a), the transmission becomes asymmetric with respect to the in-plane momentum *k*_*y *_parallel along the interface, since the magnetic field breaks the time reversal symmetry. Note that Figure 3a implies that for a certain incident angle *ϕ*, tunneling is forbidden, the boundary of the total reflection region (*T *= 0) can be approximately given by the relation *k*_*y *_+ *Bd *= *E*, which implies that the transmitted wave is an evanescent mode which decays exponentially along the propagating direction. This total reflection is always present regardless of the incidence angle *ϕ *when *E *<*Bd*/2, and results in an asymmetric behavior of the transmission as a function of the incident angle. This total reflection can be understood semiclassically: when the width *d *of the barrier is large as compared to the Fermi wavelength *λ*_*F*_, the electron inside the barrier will move on a cyclotron orbit, and if the cyclotron orbit radius *R*_*c *_<*d*, the incident electron will exit the barrier region backwards eventually. This feature illustrates that the Dirac quasi-particles can be confined by the inhomogeneous magnetic field, and, more interestingly, the incident electron spin-oriented parallel along the interface will be flipped in the total reflection region. Figure 3b shows the spin orientation as a function of the incident angles and energies, which is also asymmetric with respect to the incident angles, as a consequence of the perpendicular inhomogeneous magnetic field. Note that the transmitted electron spin no longer points along the same direction of the incident electron spin, but is rotated over an angle, which is determined by the incident angle, the electron energy and the barrier width. In addition, changing the length of the barrier and/or the magnetic field can tune the total reflection and the perfect transmission regions.

**Figure 4:**
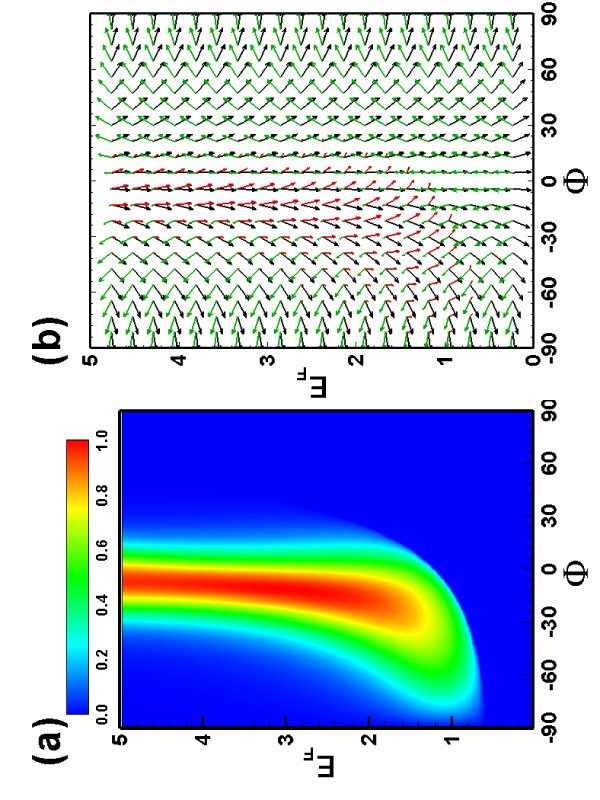
**The same as Figure 2, but now for a combined electric and magnetic barrier with *B *= 1, and *V *= 4**.

We also examined the electron transmission through a combined electric and magnetic barrier. The transmission and spin orientation (see Figure 4a,b) become very different from that of the pure magnetic barrier case. The interplay between electric barrier and magnetic field strongly reduces the perfect transmission region, and provides us with an additional way to control the transmission and the spin orientation of the transmitted and reflected electrons.

**Figure 5:**
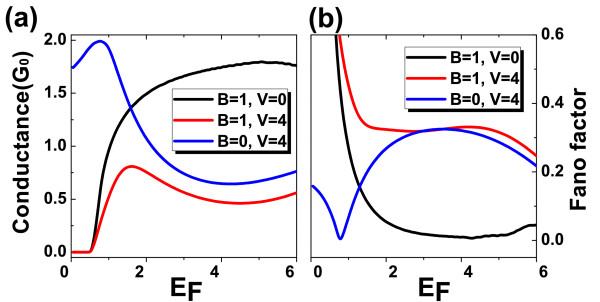
**Conductance and Fano factor of the single magnetic/electric barrier**. **(a) **The conductance as a function of the incident energy with B = 1, V = 0 (black solid line); B = 1, V = 4 (red solid line); and B = 0, V = 4 (blue solid line). **(b) **The Fano factor as a function of the incident energy for the same magnetic/electric barrier.

The transmission feature shown above can be examined by the measurable quantities, the conductance *G*, and Fano factor *F *[22,23]. The ballistic conductance and Fano factor for a given Fermi energy at zero temperature are given by summing over the modes,

(6) $$\begin{array}{c} \;\;\;\;\;G\left(E_{\text{F}}\right)=G_{0}\sum_{n=-\infty }^{\infty }T_{n}\left(E_{\text{F}}\right),\\ F\left(E_{\text{F}}\right)=\sum_{n=-\infty }^{\infty }T_{n}\left(E_{\text{F}}\right)\left[1-T_{n}\left(E_{\text{F}}\right)\right]/\sum_{n=-\infty }^{\infty }T_{n}\left(E_{\text{F}}\right), \end{array}$$

where $G_{0}\equiv \frac{e^{2}}{\pi \hbar }$ is taken as the conductance unit. The dependence of the conductance *G *and the Fano factor *F *on the incident energy *E*_F _are plotted in Figure 5. In low-energy region, the magnetic barrier can suppress the electron transmission probability for any incident angles, and thus lead to vanished conductances. The conductance increases as the Fermi energy since the magnetic field is not strong enough to confine the electrons. For a pure electric barrier, the conductance is very large in low energy region, since the electrons can effectively tunnel through the barrier via the down branch of the surface state, i.e., the Klein tunneling. When the incident energy approaches the top of an electric barrier, the conductance decrease drastically. This is because the wave vectors in the electric barrier become imaginary if the electrons are not normal incident. The Fano factor shows good correspondence with the changes of conductance. We can see clearly a significant change when the barrier changes from transparent to opaque. These quantities are what experimentalists can actually measure.

## 4. Conclusion

In summary, we investigated theoretically quantum tunneling processes through a single electric and magnetic barrier on the surface of a 3D topological insulator. Our theoretical results show that the propagating behavior of electrons in such a 2D system can be controlled by electric and magnetic barriers. We have also observed the rotation of the electron spin, depending on the parameters of the magnetic barrier structure as well as the incident angle and energy. This investigation could be helpful to offer a promising functional unit for future wavevector, and/or spin filtering quantum devices.

## Competing interests

The authors declare that they have no competing interests.

## Authors' contributions

ZW conceived of the study and carried out the numerical calculation. JL participated in establishing the physical model and developing the numerical code. All authors have participated in the interpretation of the numerical results. All authors read and approved the final manuscript.
